# IMMAN: an R/Bioconductor package for Interolog protein network reconstruction, mapping and mining analysis

**DOI:** 10.1186/s12859-019-2659-y

**Published:** 2019-02-12

**Authors:** Minoo Ashtiani, Payman Nickchi, Soheil Jahangiri-Tazehkand, Abdollah Safari, Mehdi Mirzaie, Mohieddin Jafari

**Affiliations:** 1 School of Biological Science, Institute for Research in Fundamental Sciences (IPM), Tehran, Iran; 2 Department of Statistics and Actuarial Science, Simon Fraser University, 8888 University Drive, Burnaby, BC V5A 1S6 Canada; 3 Department of Computer Science, Shahid Beheshti University, Tehran, Iran; 4 Department of Applied Mathematics, Faculty of Mathematical Sciences, Tarbiat Modares University, Tehran, Iran; 5 Institute for Molecular Medicine Finland (FIMM), Helsinki Institute of Life Science, University of Helsinki, Helsinki, Finland

**Keywords:** Protein-protein interaction networks (PPINs), Interolog protein network (IPN), Bioconductor, Network biology

## Abstract

**Background:**

Reconstruction of protein-protein interaction networks (PPIN) has been riddled with controversy for decades. Particularly, false-negative and -positive interactions make this progress even more complicated. Also, lack of a standard PPIN limits us in the comparison studies and results in the incompatible outcomes. Using an evolution-based concept, i.e. interolog which refers to interacting orthologous protein sets, pave the way toward an optimal benchmark.

**Results:**

Here, we provide an R package, IMMAN, as a tool for reconstructing Interolog Protein Network (IPN) by integrating several Protein-protein Interaction Networks (PPINs). Users can unify different PPINs to mine conserved common networks among species. IMMAN is designed to retrieve IPNs with different degrees of conservation to engage prediction analysis of protein functions according to their networks.

**Conclusions:**

IPN consists of evolutionarily conserved nodes and their related edges regarding low false positive rates, which can be considered as a gold standard network in the contexts of biological network analysis regarding to those PPINs which is derived from.

**Electronic supplementary material:**

The online version of this article (10.1186/s12859-019-2659-y) contains supplementary material, which is available to authorized users.

## Background

Nowadays, tremendous amount of interactions at the molecular level have been accessible by the development of the technology, endeavors to model cellular and molecular processes [1, 2]. Among these interactions, protein-protein interactions (PPIs) are remarkable due to providing functional and structural description of executive molecules i.e. proteins [3]. Nevertheless, PPI detection and prediction technologies are still entangling with reducing false-positive and -negative interactions [4–6]. Accordingly, data integration is the best solution overall in spite of the improvement of experimental and computational methods. STRING [7], BioNetBuilder Cytoscape app [8], IMP 2.0 [9], PINALOG [10], HIPPIE [11] and BIPS [12] are using this solution to reconstruct and refine PPI networks (PPINs). In the other works, an evolutionarily conserved network with communal nodes and less false-positive links, Interolog Protein Network (IPN), was introduced as a benchmark for the evaluation of clustering algorithms [13]. IPN clears up the arisen and remained interactions during the evolution and helps to excavate the remnants of ancestor PPIN [13–17]. In this study, we present IMMAN, a package to integrate several PPINs and mine IPNs. IMMAN is free and is available as an R/Bioconductor package and also a Java program.

## Implementation

IMMAN enables users to define two to four arbitrarily lists of proteins (by UniProt accession number) as inputs, and seek for evolutionarily conserved interactions in the integrated PPIN or IPN as the output. Briefly speaking, the method takes the following steps to accomplish this goal.

Step 1. First, the amino acid sequence of each protein of input list is automatically retrieved from UniProt database.
Step 2. In the second step, IMMAN infers the orthologous proteins. To this end, the Needleman-Wunsch algorithms is employed to compute the pairwise sequence similarities. The reciprocal best hits are retrieved and applied in the next step to increase the chance of discovering the orthologous pairs. The user can adjust different parameters of alignment algorithm as well as the sequence similarity cutoff for orthology detection.
Step 3. In this step, the nodes of the IPN are specified. Each node of the network is defined as a set of mutually orthologous proteins (OPS) such that each OPS belongs to a set of species involved in the analysis.
Step 4. In the fourth step, for each species, the PPINs are singly extracted according to the proteins constitute the OPSs or IPN nodes. The PPINs are retrieved from STRING database. Next, the user can adjust the minimal confidence score of STRING networks.
Step 5. Finally, the edges of the interolog network are extracted. To this end, for every OPS pair, the number of protein pairs (*p*_*ik*_, *p*_*jk*_) are considered such that *p*_*i*_and *p*_*j*_are connected in the PPIN of the species *k*. If this number exceeds a predefined cutoff (coverage cutoff), there would be an edge between the aforementioned nodes. The coverage cutoff can be also specified by the user to tune conservedness.

## Results

**Fig. 1:**
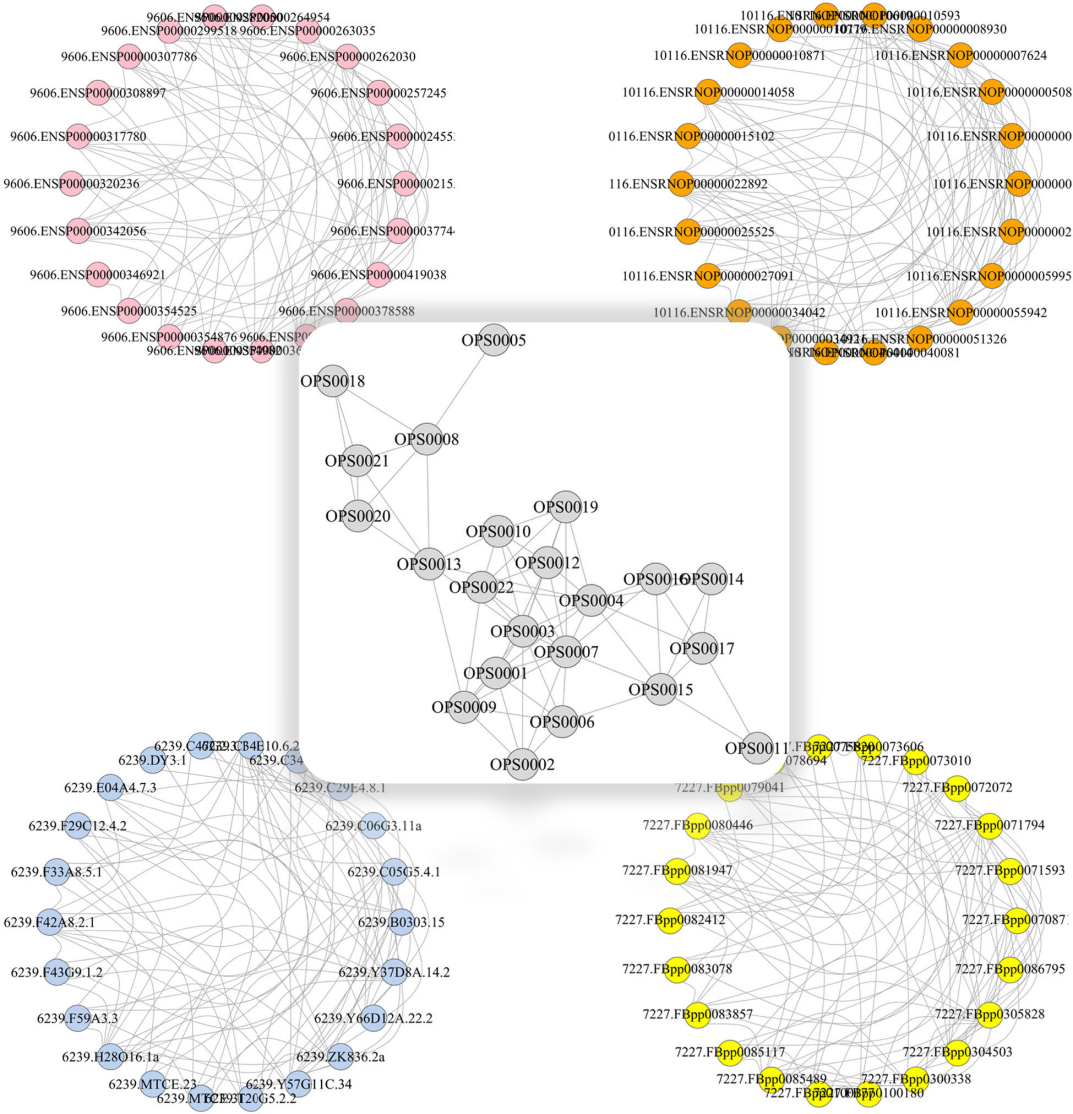
The IPN derived from four PPINs of sample species named; *H. sapiens* (top-left), *M. musculus* (top-right), *D. melanogaster* (bottom-left) and *C.elegans* (bottom-right). The size of IPN is proportional to evolutionary distance of selected species. The IPN edges is less than or equal to the smallest related PPINs. The IPN nodes are orthologous set of proteins which is abbreviated as OPS and STRINGdb IDs were used to label nodes of PPINs

After running IMMAN, the node list and the edge list of inferred IPN is produced. Additionally, IMMAN outputs the graphical representation of the network. The graphical output of IMMAN are produced using GraphViz [18] and igraph [19] in Java and R applications, respectively. The graphical representation of IMMAN on a sample dataset is depicted in Fig. 1. In this figure, the IPN derived from the original four different PPINs (Node No. ≅ 30) related to *H. sapiens*, *M. musculus*, *D. melanogaster* and *C. elegans* is represented. The resulting network contains 23 nodes and 97 to 66 edges depends on coverage parameters. Note that the higher coverage cutoff results in more stringent and conserved network. The sample dataset is available in Additional file 1.

## Conclusions

Although, the size of IPN is tunable by several thresholds, but obviously, missing the edges in IPN is the cost of true positive discovery which is an ideal within PPI studies with inherent inconsistency [6, 20]. However, function prediction is a prominent question in molecular biology and this approach pave its way based on evolutionary mechanism [21]. All routine analysis of network biology related to PPIN become more reliable by the study of IPN. For instance, finding modules within the IPN help us to understand how evolution thinks, provides and preserves cellular mechanism of species to characterize a given biological process [13]. Also, ranking the node’s influence of IPN, based on centrality measures, can shed light on the detailed mechanism of evolutionary processes [22].

## Additional file

Additional file 1: Example lists containing the UniProt accession number of four different species. (RAR 843 bytes)

## Abbreviations

IPN: Interolog Protein Network
PPI: Protein-Protein Interaction
PPIN: Protein-protein Interaction Network
